# Laparoscopic Repair of Diaphragmatic Rupture: A Case Report with Radiological and Surgical Correlation

**DOI:** 10.1155/2017/4159108

**Published:** 2017-08-20

**Authors:** Patrick Nguyen, Bonnie Davis, Daniel D. Tran

**Affiliations:** ^1^Howard University College of Medicine, Washington, DC 20059, USA; ^2^Department of Radiology, Howard University Hospital, Washington, DC 20060, USA; ^3^Department of Surgery, Howard University Hospital, Washington, DC 20060, USA

## Abstract

The leading cause of diaphragmatic rupture is penetrating abdominal trauma, including gunshot- and stab-related wounds; however, diaphragmatic rupture can also result from blunt trauma to the abdomen. The diagnosis can be difficult to make as the physical examination may be unremarkable, and imaging, that is, a conventional chest X-ray and/or CT imaging, may initially fail to reveal the injury. Failure to recognize diaphragmatic rupture can result in a delayed presentation, sometimes years later, with a potential catastrophic outcome. Therefore, prompt and swift diagnosis is critical to avoid this potential harmful scenario. Traditionally, repair is performed through a laparotomy or a thoracotomy incision. Owing to the many advances made in minimally invasive surgery, not only has laparoscopy become the modality of choice to diagnose diaphragmatic rupture due to its high degree of sensitivity and specificity, but it can provide simultaneous therapeutic intervention as well. We report a case of laparoscopic repair of a diaphragmatic rupture in a 22-year-old female who sustained blunt abdominal trauma during a motor vehicle accident.

## 1. Introduction

Diaphragmatic rupture is a rare sequelae following blunt abdominal trauma. A patient can present with a range of symptoms from a completely asymptomatic state to having severe respiratory distress. However, respiratory distress, in and of itself, can be a nonspecific presenting symptom in many other traumatic scenarios. Therefore, a high index of suspicion is necessary to make a diagnosis of diaphragmatic rupture, especially in the absence of pathognomonic radiographic findings. Although diagnostic modalities to detect diaphragmatic rupture, such as multidetector CT imaging, with a sensitivity and specificity of 61–87% and 72–100%, respectively [1], are currently in use, exploratory laparoscopy is an emerging modality in trauma care that affords greater sensitivity and specificity in diagnosing this entity, 97% and 98%, respectively [2]. Further, once a diagnosis is made with this approach, surgical repair is recommended in the same setting to prevent future complications. In recent times, an increasing number of successful laparoscopic repairs have been reported. Herein, we report a case of left diaphragmatic rupture that was successfully repaired via laparoscopy.

## 2. Case Presentation

**Figure 1:**
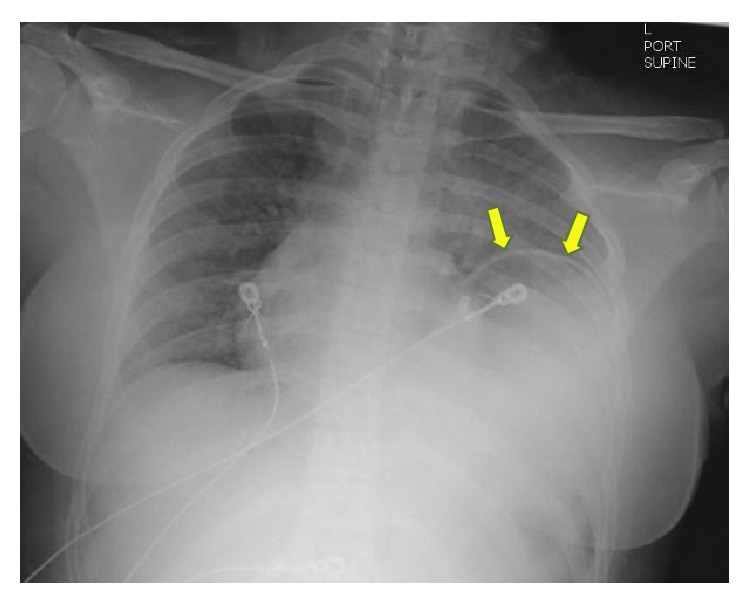
Portable supine chest radiograph shows suspected elevation of the left hemidiaphragm (yellow arrows) with underlying haziness and air-filled viscus over the lower left hemithorax. In retrospect, the suspected “elevated” diaphragmatic contour actually represents the wall of the stomach that herniated through a diaphragmatic hiatus.

**Figure 2:**
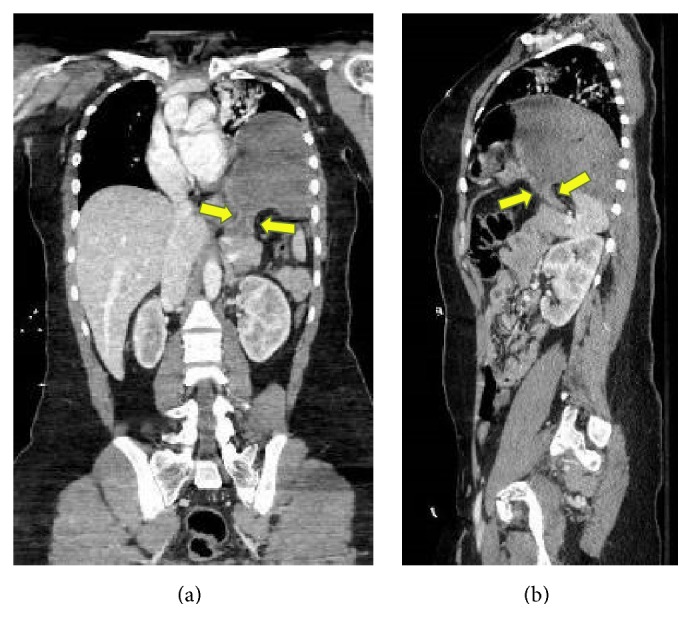
Postcontrast (intravenous) coronal (a) and sagittal (b) CT images illustrate a classic “collar” sign with focal waist-like narrowing of the gastric body (yellow arrows) as it extends from the abdominal cavity into the thorax at the site of diaphragmatic hernia.

**Figure 3:**
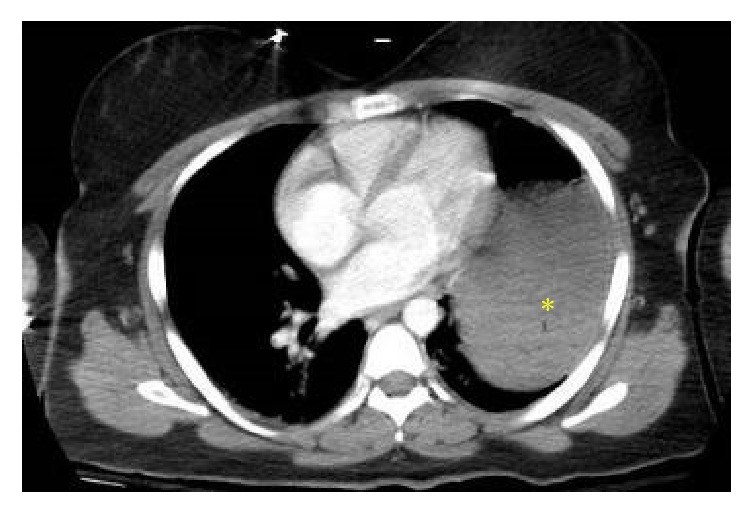
Postcontrast (intravenous) axial CT image confirms a fluid-filled viscus (yellow asterisk) in the lower left thoracic cavity.

A 22-year-old female presented to the emergency room after a motor vehicle accident complaining of abdominal and left leg pain, with a pain level described as 8/10. She was a belted, backseat passenger when the car was struck broadside. Airbags were deployed. She denied any loss of consciousness. Chest and cardiovascular examinations were unremarkable; however, she was mildly tender to deep palpation of the abdomen and could not move the left lower extremity due to pain in the left pelvis. Her work-up included a chest X-ray and CT scan of the chest, abdomen, and pelvis that revealed left lung contusion with herniation of the stomach into the left hemithorax (Figures 1–3). Aside from having mild asthma controlled with an albuterol inhaler, she lacked a significant past medical history that would suggest a diaphragmatic hernia. Additional injuries (CT images not shown) included several pelvic bone fractures, left sacroiliac joint diastasis, and a fracture of the left sacral ala.

**Figure 4:**
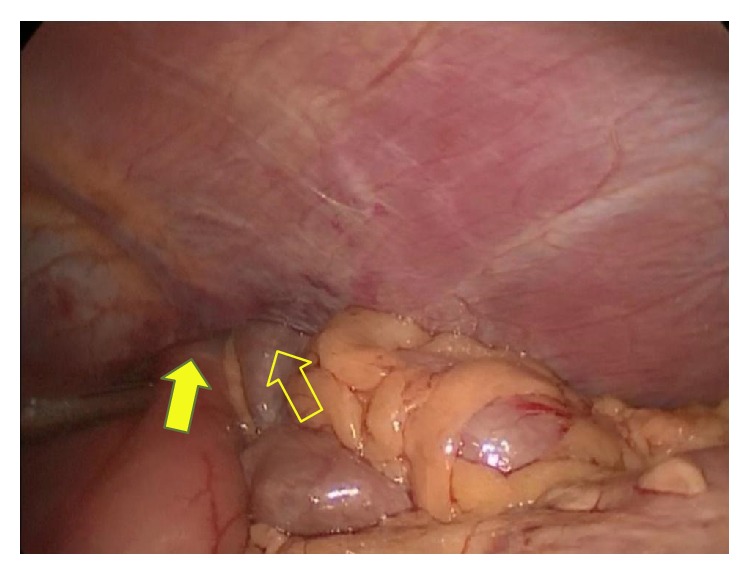
Laparoscopic view showing herniation of the stomach (yellow arrow, filled) and the transverse colon (yellow arrow, not filled) into the thorax.

**Figure 5:**
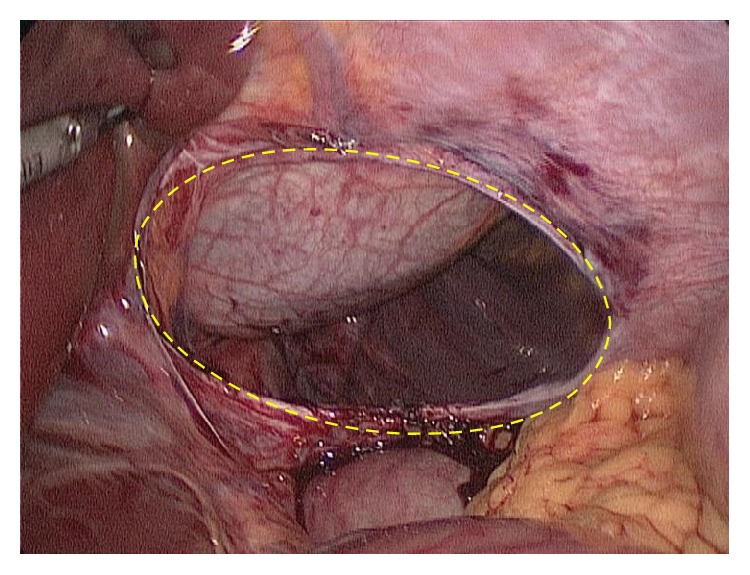
Laparoscopic view showing the diaphragmatic defect (outlined by dashed yellow oval).

**Figure 6:**
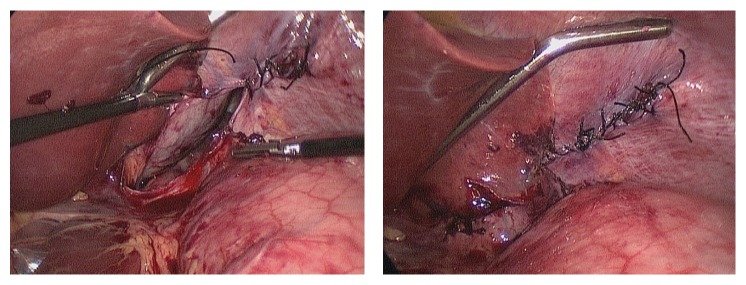
Laparoscopic view showing repair of the diaphragmatic defect.

The patient was taken to the operating room after adequate resuscitation, with a nasogastric tube already in place. The patient was intubated using a single-lumen endotracheal tube as the double-lumen tube was deemed unnecessary by the anesthesiologist. Exploratory laparoscopy was performed. Herniated bowel through a 9 cm posterior left diaphragmatic defect contained most of the stomach, loops of small bowel, and the transverse colon (Figures 4 and 5). We determined that a tension-free repair could be performed primarily without the use of mesh, with running silk suture followed by interrupted reinforcement sutures (Figure 6). At the very last stitch, prior to closure of the diaphragmatic defect, a Valsalva maneuver was performed to fully expand the lungs and expel any remaining pleural air. Therefore, there was no need for a chest tube or pleural catheter, which was ultimately confirmed by the fact that the patient never developed a pneumothorax after surgery. Upon final inspection of the abdominal cavity, there were no other signs of injury. The patient tolerated the procedure without incident and was subsequently returned to the trauma admitting unit. Following orthopedic surgical repair of her other injuries, the patient was discharged, in stable condition, five days later.

## 3. Discussion

Though penetrating and blunt trauma to the abdomen can both result in diaphragmatic rupture, penetrating abdominal trauma is the leading cause, accounting for 75% of cases [3]. The majority of ruptures occur through the left hemidiaphragm (88–95%), as opposed to the right, because of the protective effect of the liver [4]. Diaphragmatic rupture can result in herniation of intraabdominal structures, including the colon, stomach, liver, spleen, and small bowel loops [4]. Ensuing complications range from obstruction to strangulation, or ruptured viscus. Thus, early diagnosis of diaphragmatic rupture is critical.

The diagnosis, however, can be difficult because the physical examination may be unremarkable, and 30–50% of diaphragmatic ruptures are missed on initial imaging [3]. Failure to promptly recognize diaphragmatic rupture, with subsequent herniation, can result in delayed symptoms, sometimes months and even years later, with potential catastrophic outcomes [5–7]. Therefore, a high index of suspicion, especially in the face of negative imaging results, should prompt consideration of diagnostic laparoscopy, as diagnosis of diaphragmatic rupture approaches 100% when using a laparoscopic approach [8]. Laparoscopy can clearly illuminate both hemidiaphragms to look for diaphragmatic rupture and can also identify other injuries in the abdominal cavity. Furthermore, simultaneous repair of the rupture site can be performed laparoscopically, in experienced hands.

Repair of diaphragmatic rupture has been performed through a laparotomy or a thoracotomy incision for many years. However, open surgical procedures are associated with increased postoperative pain, increased hospital length of stay, and development of long-term complications, such as an incisional hernia [9, 10]. In comparison, laparoscopy offers the benefits of less postoperative pain (and thus less dependence on analgesics), faster recovery, and a decreased risk of wound complications [2, 11]. Nonetheless, in both open and minimally invasive surgical procedures, the use of mesh is necessary when the edges cannot be approximated tension-free [3]. In our case, because the diaphragmatic edges could be reapproximated without tension, primary repair was performed. Despite these advantages, there is still debate over the effectiveness of laparoscopic repair of diaphragmatic rupture following blunt abdominal trauma. Most can agree that the surgeon's expertise and careful patient selection are paramount [12, 13].

## 4. Conclusion

Diaphragmatic rupture as a result of blunt abdominal trauma is not only uncommon but also potentially underdiagnosed. In the event that there is a scarcity of indicative symptoms and radiographic findings, one must circumnavigate these limitations to reach the appropriate diagnosis. Laparoscopy is an excellent diagnostic and therapeutic procedure in the setting of suspected diagnostic rupture, with or without herniation of abdominal content. It is safe and effective in repairing the diaphragm following rupture due to blunt abdominal trauma.
